# Normothermic *Ex Vivo* Heart Perfusion with Mesenchymal Stem Cell-Derived Conditioned Medium Improves Myocardial Tissue Protection in Rat Donation after Circulatory Death Hearts

**DOI:** 10.1155/2022/8513812

**Published:** 2022-11-17

**Authors:** Zifeng Zeng, Liwei Xu, Yu Xu, Yongsheng Ruan, Deshen Liu, Jiale Li, Chuanjie Niu, Shaoyi Zheng, Pengyu Zhou, Zezhou Xiao

**Affiliations:** ^1^Department of Cardiovascular Surgery, Nanfang Hospital, Southern Medical University, Guangzhou 510000, China; ^2^Department of Pediatrics, Nanfang Hospital, Southern Medical University, Guangzhou 510000, China

## Abstract

**Objective:**

Adopting hearts from donation after circulatory death (DCD) is a promising approach to enlarge the donor pool. Nevertheless, DCD hearts experience severe warm ischemia/reperfusion (I/R) injury. Recent studies have demonstrated that conditioned medium (CM) derived from bone marrow mesenchymal stem cells (BMSCs) has the potential of reducing organ I/R injury. Therefore, we investigated whether DCD heart preservation with normothermic *ex vivo* heart perfusion (EVHP) and BMSCs-CM treatment could alleviate myocardial warm I/R injury in the DCD hearts.

**Methods:**

We randomly divided donor rats into two groups: (1) DCD-Control group and (2) DCD-CM group. Before DCD heart preservation with the normothermic EVHP system for 105 minutes, rats suffered from a 25-minute warm ischemia injury in the DCD procedure. Vehicle or CM (300 *μ*l) was added to the perfusate at the beginning of the perfusion process. The cardiac function of DCD hearts in the DCD-Control and DCD-CM groups was measured every 30 minutes. Besides, non-DCD hearts were harvested from the beating-heart rats.

**Results:**

The antibody array demonstrated that the CM contained 14 bioactive factors involved in apoptosis, inflammation, and oxidative stress. Warm ischemia injury resulted in a significant increase in the level of oxidative stress, inflammation, and apoptosis in the DCD hearts of DCD-Control group. Furthermore, compared with the DCD-Control group, CM treatment increased the developed pressure, dP/dt_max_ and dP/dt_min_ of the left ventricular in the DCD hearts during a 90-minute EVHP. Moreover, the administration of CM attenuated the level of oxidative stress, inflammation, and apoptosis in the DCD hearts of the DCD-CM group.

**Conclusions:**

Normothermic EVHP combined with CM treatment can alleviate warm I/R injury in the DCD hearts by decreasing the level of oxidative stress, inflammatory response, and apoptosis, which might alleviate the shortage of donor hearts by adopting DCD hearts.

## 1. Introduction

Heart transplantation remains the most appropriate therapy for end-stage heart failure [1]. Unfortunately, despite the increasing number of heart failure patients, the shortage of suitable donor hearts has limited the development of heart transplantation [2]. Recent studies have shown that adopting hearts from donation after circulatory death (DCD) can significantly expand the donor pool [3]. In the United Kingdom, it is predicted that adopting strict DCD donor selection criteria will result in a 56% increase in the number of heart transplantation [4], which will significantly reduce waiting-list mortality. Furthermore, compared with conventional brain-dead (BD) heart transplantation, DCD heart transplantation provided a similar 30-day or 1-year postoperative survival rate [5, 6]. However, due to inevitable warm ischemia time, DCD hearts undergo more severe myocardial ischemia/reperfusion (I/R) injury [7].

Mesenchymal stem cells (MSCs) are pluripotent, self-renewing cells [8]. The administration of MSCs is introduced as a promising cell-based therapy to alleviating organ I/R injury [9]. Moreover, the application of MSCs could significantly mitigate I/R injury during organ preservation [10]. The therapeutic efficacy of MSCs is attributed to their outstanding immunoregulatory and regenerative properties [11]. However, recent studies have demonstrated that transplanted MSCs have a relatively low long-term survival rate [12, 13], suggesting the paracrine effect of MSCs may play a significant role in their therapeutic efficacy [14]. A growing body of literature has shown condition medium (CM) derived from MSCs contains various kinds of bioactive factors, such as chemokines/cytokines [15] as well as growth and antiapoptotic factors, which can exert significant therapeutic effects on different diseases [16, 17]. Korkmaz-Icöz et al. showed preserving BD donor hearts with CM-enriched cardioplegic solution improved the post-transplant contractile properties of the grafts in rat heart transplantation [18]. Moreover, machine perfusion of donor hearts with CM protected against myocardial I/R injury in 15-month-old rats [19]. In addition, the cold preservation of vascular graft with CM alleviated vascular graft endothelial dysfunction following *in vitro* I/R injury in BD rats [20].

Static cold storage has been considered as a reliable and traditional heart preservation strategy, which is simple, inexpensive, and able to preserve standard donor hearts for 4-6 hours with acceptable transplantation outcomes [21]. However, this storage method might not be recognized as the best preservation strategy for DCD hearts, as energy-depleted DCD hearts can hardly tolerate additional cold ischemia [22]. In recent years, normothermic *ex vivo* heart perfusion (EVHP) has been recognized as a promising and novel strategy for DCD heart preservation. Normothermic EVHP can supply donated hearts with oxygenated, warm, and nutrient-enriched blood-based perfusate in a semiphysiological state during organ storage [23]. Therefore, compared with static cold storage, normothermic EVHP can attenuate myocardial I/R injury in the DCD hearts, assess the contractile function of DCD hearts in real-time, prolong storage time, and provide a unique platform for repairing DCD hearts by delivering novel drugs directly into the machine perfusion circuit [24, 25]. Until now, the cardioprotective effect of normothermic EVHP combined with CM treatment in preserving DCD hearts has not been studied .

Therefore, in the current study, we explored the cardioprotective potential of normothermic EVHP combined with CM treatment in our well-established rat model of DCD [26]. We hypothesized that the combination of normothermic EVHP and CM treatment might be a novel and promising donor heart preservation strategy to ameliorate myocardial I/R injury and improve cardiac function in the DCD hearts, thereby increasing the number of transplantable grafts in heart transplantation.

## 2. Materials and Methods

### 2.1. Animals

Male-specific pathogen-free Sprague-Dawley rats were purchased from Charles River Laboratories (Beijing, China) and acclimated for 1 week before experiments. All animals received care in compliance with the Guide for the Care and Use of Laboratory Animals (National Institutes of Health Publication No. 85-23, revised 1996). The experimental protocol was reviewed and approved by the Ethical Committee of the Laboratory Animal Research Center of Southern Medical University Nanfang Hospital. The rats were housed in consistent temperature (22 ± 2°C) rooms with a 12-12 hours dark-light cycles and were given food and sterilized water.

### 2.2. Isolation and Culture of Bone Marrow Mesenchymal Stem Cells

Bone marrow mesenchymal stem cells (BMSCs) were obtained from the bone marrow of male rats (8-week-old). Briefly, an overdose of pentobarbital sodium (100 mg/kg) was used to euthanize rats by intraperitoneal injection. To isolate bone marrow, femurs and tibias were flushed with phosphate-buffered saline (PBS, Meilunbio, Dalian, China). BMSCs were suspended in MEM Alpha basic(1X) (*α*-MEM, Gibco, Grand Island, USA) which was supplemented with 1% penicillin-streptomycin (Gibco, Grand Island, USA) and 10% fetal bovine serum (Gibco, Grand Island, USA) and then cultured at 37°C with 5% CO_2_. When 80% confluency was reached, the primary culture was subcultured at the ratio of 1 : 2. In the following experiments, only the third passage of BMSCs was used.

### 2.3. Preparation of BMSCs-CM and Perfusate

**Figure 1:**
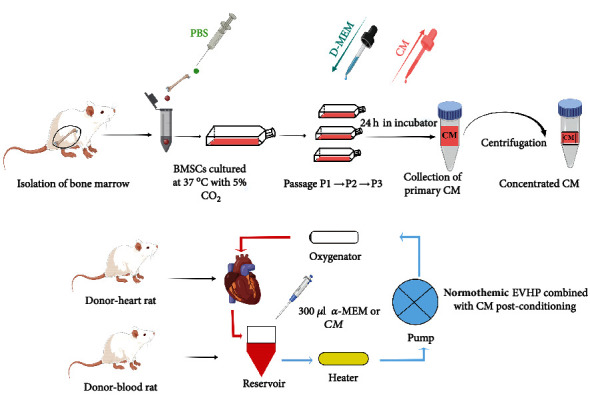
Simplified schematic image of the collection of CM from BMSCs and normothermic *ex vivo* heart perfusion combined with CM treatment. After rats were euthanized, the bone marrow was isolated by using phosphate-buffered saline (PBS) to flush the femurs and tibias. The cells were incubated on cell culture flasks at 37°C with 5% CO_2_. After BMSCs reached more than 80% confluency at passage 3, the medium was aspirated, and PBS was used to rinse the cells 3 times. Then, BMSCs were added with *α*-MEM on culture flasks in an incubator for 24 h. After primary CM was collected, concentrated CM was obtained by centrifuging primary CM with ultrafiltration units (5000 g for 2 h at 4°C). BMSCs: bone marrow mesenchymal stem cells; CM: condition medium; DCD: donation after circulatory death; EVHP: *ex vivo* heart perfusion.

A simplified schematic of the BMSCs-CM collection protocol is shown in Figure 1. After BMSCs reached more than 80% confluency at the third passage, the medium was aspirated, and then, BMSCs were washed 3 times with PBS. Then, *α*-MEM was added to culture flasks with BMSCs, and the culture flasks were put into an incubator continuously at 37°C. After incubation for 24 hours, the primary CM was collected and sequentially concentrated to 15-fold by ultrafiltration units with 5000 g for 2 h at 4°C. Finally, 300 *μ*l *α*-MEM (DCD-Control group) or BMSCs-CM (DCD-CM group) was added to the 12 ml blood-based perfusate.

### 2.4. Antibody Array

G-Series Rat Cytokine Array 67(RadBiotech, Atlanta, USA) was used to detect the relative expression of 67 target proteins in CM.

### 2.5. Experiment Design

**Figure 2:**
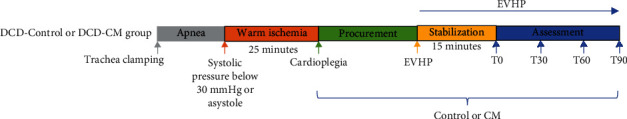
The representative experimental protocol of normothermic *ex vivo* heart perfusion combined with CM treatment in the DCD heart preservation. DCD: donation after circulatory death; EVHP: *ex vivo* heart perfusion; CM: condition medium; MP: machine perfusion.

24 male Sprague-Dawley rats (8 to 12-week-old; 200-300 g) were used as donor-heart rats, and another 24 male Sprague-Dawley rats (12 to 15-week-old; 300-400 g) were introduced as blood donors prepared for blood-based perfusate in the normothermic EVHP system. All the donor hearts were preserved by the normothermic EVHP system. Cardiac function was assessed during the EVHP period. At the end of the normothermic EVHP operation, heart tissue was collected to evaluate the level of apoptosis, oxidative stress, and inflammatory response (Figure 2).

Heart donor rats were randomly divided into 2 groups: (1) DCD-Control group and (2) DCD-CM group. Before the DCD hearts preservation with the normothermic EVHP system for 105 minutes, rats suffered from the DCD procedure and 25-minute warm ischemia injury. 20 ml cold Custodiol cardioplegic solution was used to perfuse the DCD heart. Before initiating normothermic EVHP, 300 *μ*l CM or *α*-MEM was added into 20 ml blood-based perfusate. Besides, non-DCD hearts were directly harvested from heart-beating rats, and heart tissue was immediately collected.

### 2.6. Operative Procedure

#### 2.6.1. Anesthesia

Pentobarbital sodium (60 mg/kg, intraperitoneally) was used to anesthetize Sprague-Dawley rats. Adequate anesthetic depth was determined by pedal reflex before experiments. The rats were placed on a heating pad to maintain body temperature.

#### 2.6.2. Harvest of Donor Blood

The abdominal artery was inserted by a 2-inch, 18 G I.V. catheter. The catheter was connected to a 20 ml syringe that contained 1250 IU heparin and used to withdraw 9 ml blood. The blood-based perfusate consisted of insulin (160 IU/l; Novo Nordisk; Denmark), methylprednisolone sodium succinate (500 mg/l; Pfizer; Belgium; Switzerland), mannitol (25 g/l), 6 ml modified Krebs-Henseleit solution (117 mM NaCl, 10 mM glucose, 4.5 mM KCl, 1.2 mM NaH_2_PO4, 25 mM NaHCO_3_, 0.512 mM MgCl_2_, and 2 mM CaCl_2_), and 9 ml blood withdrawn from the donor-blood rat. The blood-based perfusate was added to the perfusion circuit and then oxygenated for 15 minutes, and PaO_2_ was maintained at 150-250 mmHg.

#### 2.6.3. Harvest of Donor Heart

A 16 G, 2-inch I.V. catheter was cannulated into the trachea after tracheotomy, and the rats were mechanically ventilated. The right carotid artery was cannulated with a 22 G, 1-inch I.V. catheter for real-time blood pressure monitoring, injection of heparin, and delivery of Custodiol cardioplegic solution. Before the induction of circulatory death, heparin (2000 IU/kg body weight) was injected via the right carotid artery to heparinize the donor-heart rat.

The following DCD procedure was performed in the DCD-Control and DCD-CM groups of rats. Briefly, circulatory death was induced by clamping the trachea with mosquito forceps. The catheter which was cannulated into the right carotid artery was connected to the pressure senor. Circulatory death could be accomplished when asystole was observed or systolic pressure below 30 mmHg [24, 25]. After a 25-minute warm ischemia time (WIT, equivalent to the hands-off time), the sternotomy was performed, and the aortic arch between the left common carotid artery and the brachiocephalic trunk was clamped. After the inferior vena cava was cut and the left atrium was opened, 20 ml cold Custodiol cardioplegic solution was perfused to the DCD heart at constant pressure (60 mmHg) via the right carotid artery, and the DCD heart was put into ice. The aorta distal to the pulmonary artery and left subclavian artery was cut, respectively. The arrested heart was excised and then cannulated with a 14 G homemade aortic catheter.

#### 2.6.4. The Operation of EVHP

The normothermic ESHP system included an oxygenator (Micro-1 Rat Oxygenator; Dongguan Kewei, China), a microperistaltic pump (BT101L, Lead Fluid; China), a reservoir, 16# Tygon tubing, an infusion syringe pump (Perfusor-space; B. Braun, Germany) used for the administration of epinephrine, and a homemade water-bath box that consisted of water, stirrer, heater, aortic cannula, and temperature-controlling switch. A humidified gas mixture that contained 95% O_2_/5% CO_2_ was used to gas the oxygenator.

The detailed normothermic EVHP protocol was described before [26]. Briefly, the EVHP system was connected to the donor heart by the aortic cannula. The reservoir was partially submerged in the water of the water-bath box, and the isolated heart was put below the horizontal plane of the water. To maintain the temperature of inflow and isolated heart at 35–37°C, a thermal insulation bag was used to wrap the membrane oxygenator, and the water temperature in the box was controlled at 41–42°C. The perfusion rate of the normothermic EVHP was 2 ml/min at the beginning, and the target perfusion rate (1 ml/100 g body weight/min) was slowly reached within 10 minutes. After a 15-minute stabilization period, epinephrine (4.8 × 10^−5^ mg/kg body weight/h) was infused via an infusion syringe pump. The temperature of the isolated heart was maintained at 35-37°C and PaO_2_ at 150-250 mmHg during the normothermic EVHP period. The perfusate was supplemented by 40 *μ*l 5% sodium bicarbonate solution and 20 *μ*l 50% glucose solution every 15 minutes.

#### 2.6.5. Cardiac Functional Assessment during EVHP

At the end of the 15-minute stabilization period, the latex balloon attached to a pressure sensor was inserted into the left ventricle via the left atrium. At the beginning of assessment phase (T0), 0.15 ml of saline was slowly injected into the balloon to measure the intraventricular pressure of the donor heart every 30 minutes (T30, T60, and T90). Cardiac functional parameters of heart grafts during the EVHP period included developed pressure (systolic minus diastolic-blood pressure, DP), heart rate (HR), maximum rate of rising of left ventricular pressure (dP/dt_max_), and maximum rate of pressure decline (dP/dt_min_).

#### 2.6.6. Sample Collection

Two 1-2 mm thick ventricle slices were collected sequentially along the long axis after a 105-minute EVHP. The first myocardial tissue taken from the apex was used for Western blotting and the second one for immunohistochemical and histologic analysis.

#### 2.6.7. Oxidative Stress

Immunohistochemical analysis was performed to determine the expression of 4-hydroxynonenal (HNE) as an indicator of oxidative stress.

#### 2.6.8. Inflammatory Response

The expression of tumor necrosis factor-*α* (TNF-*α*), nuclear factor kappa-B p65 (NF-*κ*Bp65), and interleukin-6 (IL-6) was measured by immunohistochemistry. The expression of myeloperoxidase (MPO) was analyzed by Western blotting.

#### 2.6.9. Immunohistochemistry

After being fixed in paraformaldehyde solution (4%), myocardial tissue was embedded in paraffin and then cut into 4-*μ*m-thick sections. The immunoreactivity to TNF-*α* (1 : 200, Abcam, ab109322, USA), IL-6 (1 : 500, Abcam, ab9324, USA), HNE (1 : 1000, Abcam, ab46545, USA), and NF-*κ*B p65 (1 : 500, Proteintech, 66535, China) was assessed. Diaminobenzidine reaction visualized the antigen-antibody reaction. The fields of each slice were randomly chosen and recorded blindly under a conventional light microscope. Image-Pro Plus software (Media Cybernetics, USA) was used to perform image analysis. The assessment was performed in four nonoverlapping and random fields of the heart tissue. The mean value was calculated for each animal. The expression of TNF-*α*, IL-6, HNE, and NF-*κ*B p65 was measured by counting integrated optical density (IOD).

#### 2.6.10. Apoptosis

DNA-strand breaks in the donor hearts were detected by terminal deoxynucleotidyl transferase dUTP nick end labeling (TUNEL) staining. The number of TUNEL-positive cells was counted under the fluorescence microscope. The apoptosis rate of myocardium was represented by the ratio of 4′,6-diamidino-2-phenylindole (DAPI)-TUNEL double-labeled nuclei to the total number of nuclei stained with DAPI. The expression of Cleaved caspase-3, B-cell lymphoma-2 (Bcl2), and Bcl2-associated X protein (Bax) was measured by Western blotting.

#### 2.6.11. Western Blotting

The expression of the targeted protein in the donor heart was evaluated by Western blotting. The expression of MPO (1 : 250 dilution, ab65871, Abcam, USA), Cleaved caspase-3 (1 : 1000 dilution, 9664, Cell Signaling Technology (Shanghai) Biological Reagents Company Limited, China), Bcl2 (1 : 4000 dilution, 60178, Proteintech, USA), Bax (1 : 8000 dilution, 60267, Proteintech, China), and Cytochrome c (1 : 1000 dilution, 146F3, Cell Signaling Technology (Shanghai) Biological Reagents Company Limited, China) was evaluated.

#### 2.6.12. Statistical Analysis

The results were expressed as mean ± standard error of the mean (SEM). Statistical analysis was performed via GraphPad Prism 9.0 software (GraphPad Software, Inc., San Diego, CA, USA). The normality of data was tested by the Shapiro-Wilk test before statistical tests were applied. Multiple comparisons were performed using one-way ANOVA followed by Tukey's post hoc test. If the data failed the normality test, the nonparametric Kruskal-Wallis test followed by Dunn's post hoc test was used. Two-way ANOVA analysis was conducted to compare intraventricular pressure measurement recordings at different time points between DCD-Control and DCD-CM groups. A value of *p* < 0.05 was considered statistically significant.

## 3. Results

### 3.1. Protein Array-Based CM Characterization

**Figure 3:**
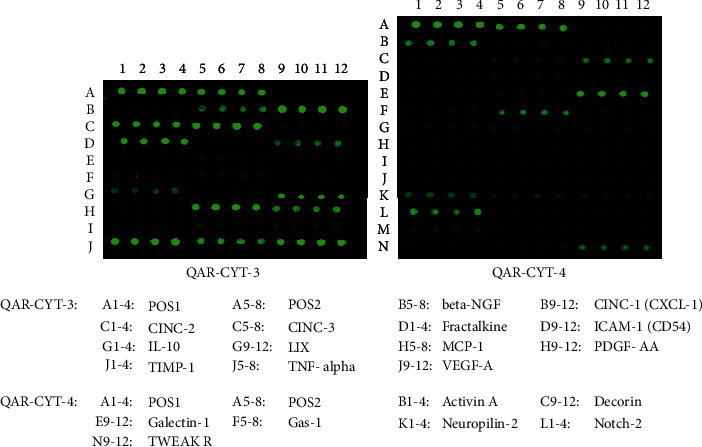
Characterization of CM by antibody-based protein array.

Antibody-based protein array analysis demonstrated that soluble bioactive factors secreted by BMSCs included adhesion molecules, cytokines/chemokines, growth factors, and other proteins (Figure 3). Among them, at least 14 bioactive factors were involved in oxidative stress, apoptosis, and inflammation (Table 1).

### 3.2. Normothermic EVHP Combined with CM Treatment Improved the Cardiac Function Damage in the DCD Heart

**Figure 4:**
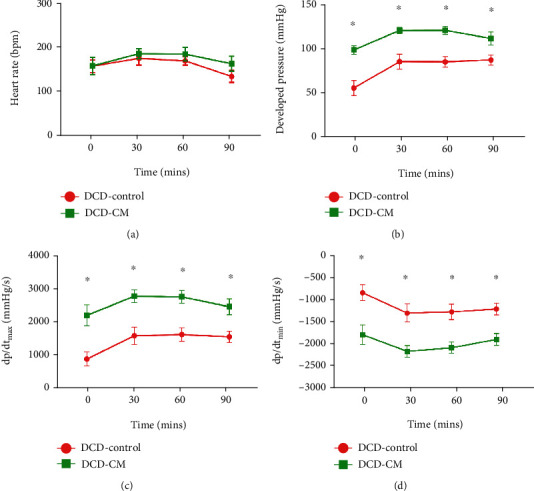
Assessment of the cardiac function of the DCD heart during EVHP period. (a) Heart rate, (b) developed pressure, (c) dP/dt_max_, and (d) dP/dt_min_. Data represent the mean ± standard error of the mean. *n* = 8 for each group. dP/dt_max_, maximum rate of rising of the left ventricular pressure. dP/dt_min_, maximum rate of pressure decline. ^∗^*p* < 0.05 vs. DCD-Control, ^#^*p* < 0.05 vs. non-DCD.

As Figure 4 shown, compared with the DCD-Control group, the addition of BMSCs-CM into the EVHP system led to a significant increase in DP (from T0 to T90, Figure 4(b)), dP/dt_max_ (from T0 to T90, Figure 4(c)), and dP/dt_min_ (from T0 to T90, Figure 4(d)), which indicated that CM treatment could protect the cardiac function of the DCD hearts from warm ischemia/reperfusion injury. However, there was no significant difference in heart rate between both groups (from T0 to T90, Figure 4(a)).

### 3.3. Normothermic EVHP Combined with CM Treatment Attenuated Oxidative Stress and Inflammation in the DCD Heart

**Figure 5:**
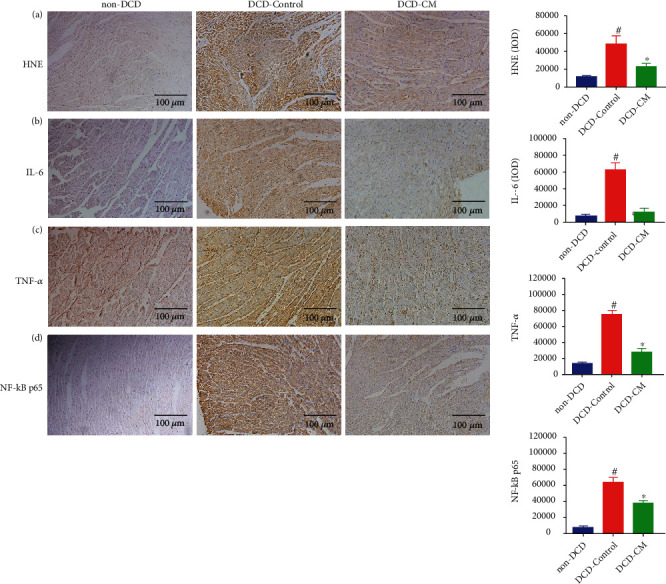
The representative photomicrographs and quantitative analysis of oxidative stress and inflammation in the DCD heart. The expression of (a) HNE, (b) IL-6, (c) TNF-*α*, and (d) NF-*κ*B p65 in the DCD heart. Data represent the mean ± standard error of the mean. *n* = 6 for each group. HNE: 4-hydroxynonenal; IL-6: interleukin-6; IOD: integrated optical density; TNF-*α*: tumor necrosis factor-*α*; NF-*κ*B: nuclear factor kappa-B. ^∗^*p* < 0.05 vs. DCD-Control, ^#^*p* < 0.05 vs. non-DCD.

**Figure 6:**
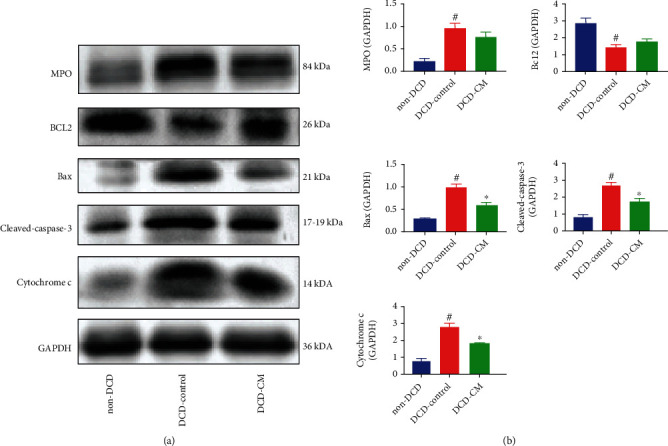
Representative protein band densities and quantitative analysis of inflammation and apoptosis in the DCD heart. (a) Representative protein band densities of MPO, Bcl2, Bax, Cleaved caspase 3, and Cytochrome c and (b) quantitative analysis of protein expression. Data represent the mean ± standard error of the mean. *n* = 4 for each group. MPO: myeloperoxidase; Bcl2: B-cell lymphoma-2; Bax: Bcl2-associated X protein. ^∗^*p* < 0.05 vs. DCD-Control, ^#^*p* < 0.05 vs. non-DCD.

To evaluate the inflammatory response in the myocardium, the expression of HNE, IL-6, TNF-*α*, and NF-*κ*B in myocardial tissue was detected by immunohistochemical method, and the expression of MPO was analyzed by Western blotting. Compared with the non-DCD hearts, the expression of HNE (Figure 5(a)), IL-6 (Figure 5(b)), TNF-*α* (Figure 5(c)), NF-*κ*B (Figure 5(d)), and MPO (Figure 6) significantly increased in the DCD-Control group, implying that warm ischemia/reperfusion injury could increase the level of myocardial apoptosis, oxidative stress, and inflammation in the DCD hearts. However, compared with the DCD-Control group, CM treatment significantly decreased the expression of HNE, IL-6, TNF-*α*, and NF-*κ*B in the DCD hearts in the DCD-CM group (Figure 5). However, no significant difference was observed in the expression of MPO between the DCD-Control and DCD-CM groups (Figure 6).

### 3.4. Normothermic EVHP Combined with CM Treatment Reduced Myocardial Apoptosis in the DCD Heart

**Figure 7:**
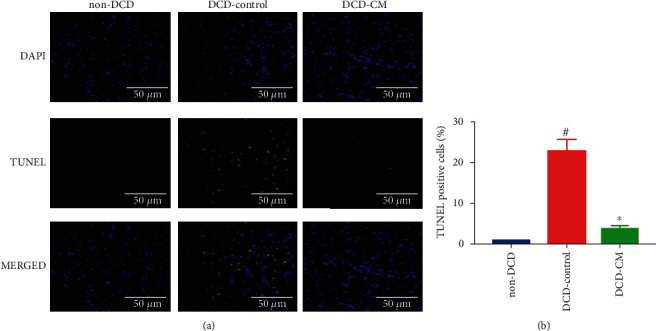
The evaluation of apoptosis in the DCD heart. (a) Representative photomicrographs of myocardial tissue stained with DAPI (blue), nuclei with fragmented DNA shown by TUNEL staining, and merged image (magnification of 40, scale length: 100 *μ*m) and (b) quantification of TUNEL-positive cells (as a percentage). Data represent the mean ± standard error of the mean. *n* = 6 for each group. DAPI, 4′, 6-diamino-2-phenylindole (DAPI, blue). TUNEL: terminal deoxynucleotidyl transferase-mediated dUTP nick end labeling. ^∗^*p* < 0.05 vs. DCD-Control, ^#^*p* < 0.05 vs. non-DCD.

TUNEL staining was performed to assess the apoptosis of myocardium. The expression of Cleaved caspase-3, Bax, Cytochrome c, and Bcl2 was detected by Western blotting. As Figures 6 and 7 shown, compared with the non-DCD hearts, the number of TUNEL-positive nuclei and the expression of Cleaved caspase-3, Bax, and Cytochrome c significantly increased, and the expression of Bcl2 significantly decreased in the DCD-Control group. However, compared with the DCD-Control group, CM treatment significantly decreased the number of TUNEL-positive nuclei and the expression of Cleaved caspase-3, Bax, and Cytochrome c, which suggested that CM treatment attenuated myocardial apoptosis in the DCD hearts. No significant difference was found in the expression of Bcl2 between DCD-Control and DCD-CM groups.

## 4. Discussion

The present study demonstrated that normothermic EVHP combined with CM treatment could be a novel and promising DCD heart preservation strategy, which could alleviate myocardial I/R injury in rat DCD hearts, as indicated by the improved heart function during EVHP period, reduced level of myocardial apoptosis, inflammation, and oxidative stress in the DCD hearts after EVHP period.

In present, DCD heart transplantation has received a great deal of attention over the past 15 years [27] and this promising clinical strategy can significantly expand the donor pool [28]. However, the use of DCD hearts is hindered by severe I/R injury as a result of the inherent warm ischemia injury [29]. Normothermic EVHP is emerging as an attractive and pioneering strategy for the preservation of the DCD hearts. Unlike traditional static cold storage method, normothermic EVHP can preserve the donated heart in a perfused, semiphysiologic state [30], which can provide a thorough assessment of donor heart viability [31] and the recondition of donor heart during machine perfusion with stem cells, gene, and pharmacological therapy [32]. In line with this novel theory, our previous study [13] demonstrated that normothermic EVHP combined with melatonin treatment attenuated myocardial I/R injury in the DCD hearts by inhibiting NLRP3 inflammasome-mediated pyroptosis. Therefore, normothermic EVHP combined with novel pharmacological agent treatment is expected to make more and more DCD hearts transplantable.

Recent studies have shown that the preservation of CM-enriched cardioplegic solution alleviates myocardial I/R injury and improves posttransplant cardiac function of the donor hearts in a rat heart transplantation model [18, 19, 33]. BMSCs release numerous kinds of bioactive factors, including chemokines, bioactive lipids, metabolites, cytokines, and growth factors, which may account for some of the cardioprotective effects observed. In the current study, the characterization of CM by antibody-based protein array analyses indicated that Decorin (DCN), activin A, Galectin-1, TIMP-1, and VEGF-A might be involved in the improved cardioprotection and cardiac function observed.

Decorin, a proteoglycan component of the extracellular matrix (ECM), possesses potent anti-inflammatory, antifibrotic, antiangiogenic, and antioxidant properties [34]. It has been shown that decorin may exert cardioprotective effects against I/R injury [35]. In addition, Oshima et al. demonstrated that activin A inhibited cardiomyocyte apoptosis induced by acute ischemia and hypoxia [36]. Furthermore, Galectin-1 was recognized as a pivotal mediator of cardiovascular homeostasis due to its prominent anti-inflammatory, proangiogenic, and procatabolic activities [37]. TIMP1 is a regulator of angiogenesis, cell apoptosis, and proliferation [38]. Singla and McDonald and Glass and Singla showed that TIMP-1 derived from embryonic stem cells improved cardiac remodeling by inhibiting cardiomyocyte apoptosis following myocardial infarction [39, 40]. Moreover, Braile et al. found that VEGF-A played an important role in reparative wound healing of the myocardium by recruiting the stem cells and decreasing myocardial apoptosis [41]. In accordance with these previous studies, the present data showed that the cocktail effect of soluble bioactive factors identified in CM might attenuate I/R injury and improve the cardiac function of DCD hearts during EVHP period, suggesting that normothermic EVHP combined with CM treatment could be an effective DCD heart preservation strategy. However, further investigation is warranted to elucidate the role of other bioactive factors identified in CM derived from BMSCs against I/R injury in the DCD hearts.

In heart transplantation, cold ischemia and warm reperfusion injury inevitably occur in the donor heart, which can lead to PGD in the transplanted heart. I/R injury is a key pathological process affecting the clinical outcomes of heart transplantation [42] due to the absence of ATP, intracellular calcium overload, inflammatory cytokines, the generation of ROS, and oxidative stress [43, 44]. Oxidative stress, inflammation, and apoptosis are thought to be involved in the pathogenesis of I/R injury, resulting in tissue damage to the donor heart. Caspases are important mediators of apoptosis. The activation of caspase-3 (the presence of its cleaved form) is a reliable indicator of the apoptosis rate, which can result in the cleavage of protein substrates and the apoptotic DNA fragmentation [45]. In the present study, a 25-minute warm ischemia injury significantly increased the level of oxidative stress (increased expression of HNE), inflammation (increased expression of TNF-*α*, IL-6, NF-*κ*B, and MPO), and apoptosis (increased expression of Bax, Cleaved caspase-3, Cytochrome c, and DNA strand breaks, and decreased expression of Bcl2) in the DCD hearts. However, compared with the DCD-Control group, normothermic EVHP combined with CM treatment decreased the level of oxidative stress (decreased expression of HNE), inflammation (decreased expression of TNF-*α*, IL-6, and NF-*κ*B), and apoptosis (decreased expression of Bax, Cleaved caspase-3, Cytochrome c, and DNA strand breaks) in the DCD heart after EVHP. Therefore, normothermic EVHP combined with CM treatment could protect the DCD heart against I/R injury-induced graft dysfunction via the inhibition of oxidative stress, inflammation, and apoptosis.

Several limitations in the present study need to be clarified. Firstly, the concentration of CM in the perfusate of the EVHP system may not be optimal in the context of DCD heart preservation. Therefore, it is necessary to design different concentrations of CM ensuring a more suitable concentration in the future research. Secondly, it would be more clinically relevant to investigate the post-transplant cardiac function of the DCD heart in a rat heart transplantation model. Thirdly, the present study could not precisely identify the signaling pathways or specific cytokines responsible for the cardioprotective effect of CM in the preservation of DCD hearts. To fully understand its exact mechanism, future experimental research should be carried out by inhibiting or overexpressing some of the specific cytokines or signaling pathways.

## 5. Conclusions

In conclusion, the present study reveals that normothermic EVHP combined with CM treatment significantly attenuates cardiac dysfunction against I/R injury in rat DCD hearts, partially by reducing oxidative stress, apoptosis, and inflammatory response. In view of clinical point, the combination of normothermic EVHP and CM treatment could be a novel and promising DCD heart preservation strategy, thereby increasing the number of transplantable grafts in heart transplantation.

## Figures and Tables

**Table 1:** Bioactive factor detected in CM from BMSCs.

Phenomena	Specific properties	Factors
Inflammatory	Proinflammatory	CINC1/2/3, MCP1, TNF-*α*, TWEAK R
	Anti-inflammatory	IL-10, Decorin, Galectin-1
Stress	Oxidative stress	Decorin
Apoptosis	Antiapoptotic	TIMP1, Activin A, VEGF-A, Decorin
Others	Immunomodulator	LIX, IL-10
	Chemokines	Fractalkine
	Adhesion molecules	ICAM-1
	Proangiogenesis	MCP1, PDGF-AA, CINC1
		VEGF-A, Neuropilin-2
		TWEAK R, Notch-2
	Cell proliferation	TIMP1, *β*-NGF, Notch-2
	ECM modulation	TIMP1

CINC1/2/3: cytokine-induced neutrophil chemoattractant 1/2/3; MCP1: monocyte chemoattractant protein-1; TNF-*α*: tumor necrosis factor-*α*; TWEAK R: TNF-like weak inducer of apoptosis R; IL-10: interleukin-10; TIMP1: tissue inhibitor of metalloproteinase-1; VEGF-A: vascular endothelial growth factor-A; LIX: lipopolysaccharide-induced chemokine; ICAM-1: intercellular cell adhesion molecule-1; MCP-1: monocyte chemoattractant protein-1; PDGF-AA; platelet-derived growth factor-AA; *β*-NGF: *β*-nerve growth factor.

## Data Availability

The raw data supporting the conclusions of this article will be made available by the authors, without undue reservation.
